# Electric nets and sticky materials for analysing oviposition behaviour of gravid malaria vectors

**DOI:** 10.1186/1475-2875-11-374

**Published:** 2012-11-14

**Authors:** Sisay Dugassa, Jenny M Lindh, Steve J Torr, Florence Oyieke, Steven W Lindsay, Ulrike Fillinger

**Affiliations:** 1 Centre of Insect Physiology and Ecology-Thomas Odhiambo Campus, Mbita, Kenya; 2 University of Nairobi, Nairobi, Kenya; 3 Royal Institute of Technology, Stockholm, Sweden; 4 Natural Resources Institute, University of Greenwich, Greenwich, UK; 5 Department of Disease Control, London School of Hygiene & Tropical Medicine, London, UK; 6 Durham University, Durham, UK

**Keywords:** Malaria, *Anopheles gambiae*, Oviposition, Electric nets, Sticky film

## Abstract

**Background:**

Little is known about how malaria mosquitoes locate oviposition sites in nature. Such knowledge is important to help devise monitoring and control measures that could be used to target gravid females. This study set out to develop a suite of tools that can be used to study the attraction of gravid *Anopheles gambiae s.s.* towards visual or olfactory cues associated with aquatic habitats.

**Methods:**

Firstly, the study developed and assessed methods for using electrocuting nets to analyse the orientation of gravid females towards an aquatic habitat. Electric nets (1m high × 0.5m wide) were powered by a 12V battery via a spark box. High and low energy settings were compared for mosquito electrocution and a collection device developed to retain electrocuted mosquitoes when falling to the ground. Secondly, a range of sticky materials and a detergent were tested to quantify if and where gravid females land to lay their eggs, by treating the edge of the ponds and the water surface. A randomized complete block design was used for all experiments with 200 mosquitoes released each day. Experiments were conducted in screened semi-field systems using insectary-reared *An. gambiae s.s.* Data were analysed by generalized estimating equations.

**Results:**

An electric net operated at the highest spark box energy of a 400 volt direct current made the net spark, creating a crackling sound, a burst of light and a burning smell. This setting caught 64% less mosquitoes than a net powered by reduced voltage output that could neither be heard nor seen (odds ratio (OR) 0.46; 95% confidence interval (CI) 0.40-0.53, p < 0.001). Three sticky boards (transparent film, glue coated black fly-screen and yellow film) were evaluated as catching devices under electric nets and the transparent and shiny black surfaces were found highly attractive (OR 41.6, 95% CI 19.8 – 87.3, p < 0.001 and OR 28.8, 95% CI 14.5 – 56.8, p < 0.001, respectively) for gravid mosquitoes to land on compared to a yellow sticky film board and therefore unsuitable as collection device under the e-nets. With a square of four e-nets around a pond combined with yellow sticky boards on average 33% (95% CI 28-38%) of mosquitoes released were collected. Sticky materials and detergent in the water worked well in collecting mosquitoes when landing on the edge of the pond or on the water surface. Over 80% of collected females were found on the water surface (mean 103, 95% CI 93–115) as compared to the edge of the artificial pond (mean 24, 95% CI 20–28).

**Conclusion:**

A square of four e-nets with yellow sticky boards as a collection device can be used for quantifying the numbers of mosquitoes approaching a small oviposition site. Shiny sticky surfaces attract gravid females possibly because they are visually mistaken as aquatic habitats. These materials might be developed further as gravid traps. *Anopheles gambiae* s.s. primarily land on the water surface for oviposition. This behaviour can be exploited for the development of new trapping and control strategies.

## Background

Indoor-resting populations of malaria vectors declined in many African countries with the massive scale-up of long-lasting insecticidal nets and indoor residual spaying [1,2]. This is due not only to the mortality of mosquitoes that contact the insecticides but also due to their behavioural avoidance of contaminated surfaces [2-8]. In areas where malaria transmission occurs outdoors at low densities [9,10], light traps and other indoor surveillance tools, may underestimate transmission. Consequently, there is need to develop novel surveillance and control tools targeting vector populations outdoors [8,11-14]. Sampling of gravid females may provide better opportunities to quantify the size of the vector population, and may be an approach that is more acceptable to local communities since monitoring does not require entering a house.

The rational development of such tools is dependent on an understanding of the behaviour and ecology of vectors [15]. For instance, extensive studies of the processes involved in host seeking in *Anopheles gambiae s.l.* led to the development of a set of highly effective intervention strategies targeting indoor resting and feeding populations [15-17]. Similarly, an improved knowledge of how mosquitoes select an aquatic habitat in which to lay their eggs might provide the basis for new control strategies that exploit oviposition behaviour of *Anopheles*. For several culicine and aedine disease vectors, an understanding of oviposition behaviour has led to effective monitoring techniques and intervention strategies [18-22]. By contrast, surprisingly little is known about the oviposition behaviour in *An. gambiae* s.l.*,* the major malaria vector in sub-Saharan Africa. As a consequence, methods for monitoring and control exploiting this behaviour are poorly developed.

To analyse oviposition behaviour, methods are needed to quantify the flight, landing and egg-laying behaviour of gravid mosquitoes in the wild. Two approaches offer the prospect of being suitable. First, electric nets (e-nets) have been used to study the orientation and landing responses of insects towards visual and chemical cues [23-28]. They were originally developed by Vale [23] to study the behaviour of tsetse flies and have been widely used to study odour- and trap-oriented behaviours of these flies [24-26,29,30]. Whilst e-nets have been used to study the behaviour of host-seeking mosquitoes [27,28], there is no report of them being used for studying the behaviour of gravid malaria vectors. Second, surfaces coated with adhesive have also been widely employed to sample insects as they land on a surface [31-39] and this approach might be used to sample mosquitoes as they land. These traps are cheap, work without a battery and, providing the adhesive is sufficiently strong, will prevent trapped insects from being eaten by most common predators. Third, adding surfactants (e.g. detergents) to the water to reduce surface tension, insects can be sampled as they land on water [40].

The present study was carried out to explore the use of electric nets and sticky materials for analysing oviposition behaviour of gravid *An. gambiae* s.s. This study set out to develop a set of tools that can be used to study the attraction of gravid *An. gambiae* s.s*.* towards visual or olfactory cues associated with aquatic habitats. Specifically, the aim was to bisect the behaviour into two components: (1) approaching an aquatic habitat and (2) the actual process of egg-laying.

## Methods

### Study site

The study was carried out a semi-field system [41] located at the International Centre of Insect Physiology and Ecology, Thomas Odhiambo Campus (icipe-TOC), Mbita, on the shores of Lake Victoria, Kenya (geographic coordinates 0° 26’ 06.19” S, 34° 12’ 53.13”E; altitude 1,137m above sea level). This area is characterized by a very consistent tropical climate with an average minimum temperature of 16°C and an average maximum temperature of 28°C (based on data from icipe-TOC meteorological station for 2010–2011). The area experiences two rainy seasons, the long rainy season between March and June and the short, and less reliable rainy season between October and December. The average annual rainfall for 2010–2011 was 1,477mm.

### Semi-field systems

**Figure 1:**
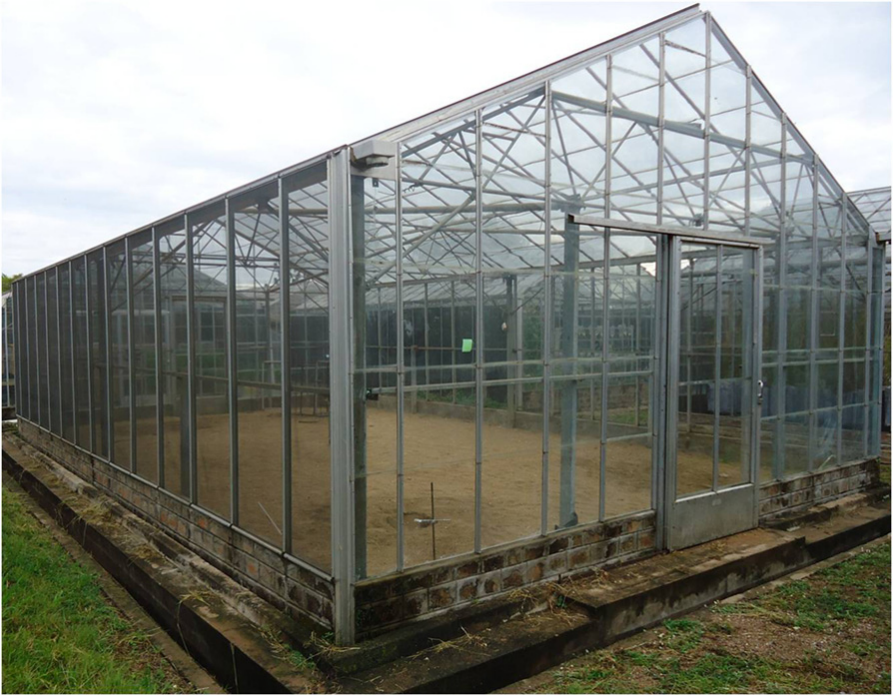
**The semi-field system at icipe-Thomas Odhiambo Campus, Mbita, Kenya**.

**Figure 2:**
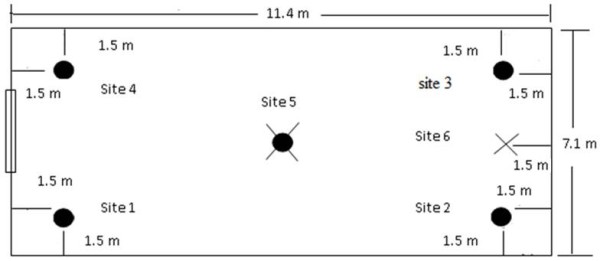
**A schematic drawing of the semi-field system with the treatment sites and release points.** X: mosquito release sites, round black circles: location of treatment.

The semi-field system was a screened greenhouse-like building (Figure 1) 7.1m wide, 11.4m long and 2.8m high at the wall and 4.0m high at the highest point of the roof [15]. The two opposite shorter walls and the roof were made of glass and the two longer walls were screened with black fibreglass netting gauze (1.7×1.5mm). The floor was covered with sand to a depth of 30cm so that artificial aquatic habitats could be dug in to the ground to simulate a natural breeding site for the mosquitoes. To increase the relative humidity inside the semi-field system to 60-70% for experiments the sand floor was watered from 15:00–16:00h prior to the experiment. Care was taken to ensure that no pooling of water occurred on the floor and that the upper layer of sand was dry by the time mosquitoes were released into the system. When treatments were positioned in the corners of the semi-field system (Site 1–4) mosquitoes were released in the centre and when the treatment was positioned in the centre (Site 5) mosquitoes were released 1.5m from the wall at Site 6 (Figure 2). Treatments in the corners were always placed 1.5m from the two adjacent walls.

### Artificial aquatic habitats

Two types of artificial habitats were used for experiments. For most experiments round ponds were constructed by positioning a black plastic bowl of 15L capacity (36cm diameter and 18 cm depth) into the ground so that the upper lip was at the same level as the sand floor. The pond was then filled with 9L of water originating from Lake Victoria and filtered through a charcoal-sand filter [42] henceforth called filtered lake water. Rectangular ponds were constructed by positioning black plastic containers of 17Lvolume (50cm long, 37cm wide and 18cm deep) into the ground and filled with 9L of filtered lake water.

### Electric nets (e-nets)

**Figure 3:**
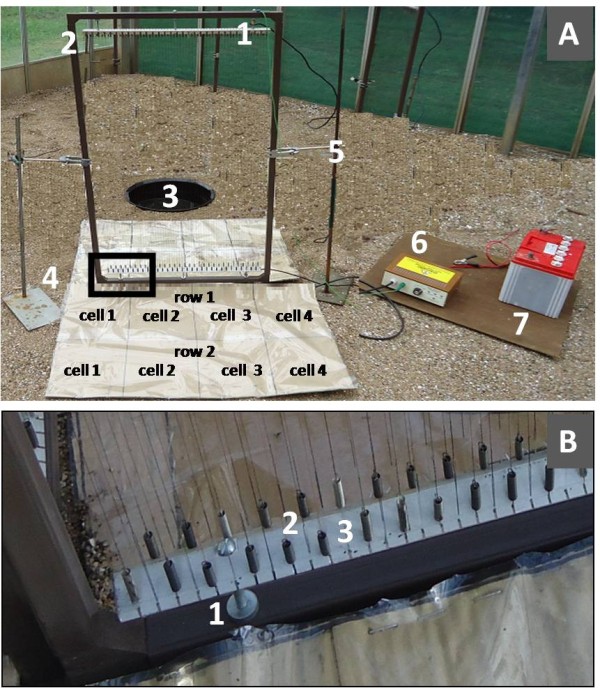
**Electrocuting net with two mosquito collection boards made of transparent sticky film.** (**A**) Overview of the set up: (1) aluminium rod, (2) aluminium frame, (3) artificial pond, (4) sticky boards on both sides of e-net, (5) stand and clamp to hold e-net, (6) spark box, (7) 12V battery. (**B**) Detail of wire connections: (1) bolt, (2) spring, (3) loop of fish line.

E-nets consist of high-tension wires stretched in parallel, across an aluminium frame (1.0 m high×0.5 m wide) with aluminium rods fixed to the two shorter opposite sides of the frame (Figure 3A). Electricity flows between the two ends of each wire generating differentials of >2.5 kV between adjacent wires [23,27], which kills insects that touch the wires. The wires are invisible to flying insects and do not have significant impact on air movement [29].

The rods had holes at a distance of 8mm for fixing the wires into the rods, to enable the electric wires to be arranged in a vertical position. Small nylon loops (Damyl® fishing lines) were tied of the same size as Fabory® zinc-plated draw springs (0.5×3.5×20mm). Copper wires (diameter of 0.2mm) 1m in length were tied to the fish line loops (insulator) from one end and to the springs (conductors) on the other end (Figure 3B). The ends of the wires with the springs and with the fish lines were alternately fitted to the holes 8mm apart on rods to enable the flow of opposite charges in opposite directions (Figure 3A). Torr *et al.*[28] assessed different spacing of wires in the electric nets and observed no difference in mosquito catch size between 4, 6 and 8mm spacing. The e-nets were held upright by using clamps on metal stands with base (Figure 3A). Alternate wires in each row were charged by a 12V car battery via a transformer (spark box). In the spark box, the 12V direct current (DC) is first converted to an alternating current (AC) that is stepped up to 400 volts peak AC. It is then rectified and converted back to 400V DC. The 400V DC voltage is used to charge a bank of capacitors that are then discharged into the primary of the ignition coil. The voltage output to the e-nets can be reduced by an energy dial lowers the 12 DC input to the spark box, that in turn lowers the 400V output that charges the capacitors. The dial position roughly equates to the energy reduction not to a direct conversion of the voltage outputs to the nets. The output is 400V at 100% spark energy setting and approximately 300V at the 50% spark energy setting of the dial.

### Sticky materials and detergent

A range of different materials were used as trapping devices for mosquitoes in the experiments. Throughout the manuscript reference is made to the materials listed in Table 1.

**Table 1:** Reference list of materials used in the experiments

**Common name used**	**Product name**	**Manufacturer**
Transparent double-sided sticky film	Clear rollertrap	Oecos, UK
Yellow sticky film	Yellow rollertrap	Oecos, UK
Transparent sticky film	FICSFIL	Barrettine, UK
Insect glue	OecoTak A5	Oecos, UK
Spray glue	Oecos spray	Oecos, UK
Detergent	Teepol	Chemical Industries, Nairobi
Black fly-screen	Polyester coated fibreglass mosquito netting (15×17 holes/ 2.54 cm^2^)	Polytrex, China
Wire screen	Dark-green wire screen (9×11 holes/ 2.54 m^2^)	Hebei Jimano, China
Transparency	A4 overhead projector transparency film (0.1 mm)	Ryman, UK

### Mosquito preparation

Insectary-reared *An. gambiae* s.s. mosquitoes were used throughout. Gravid mosquitoes were prepared as follows;300 female and 300 male mosquitoes, two to three days old, were kept in 30×30×30cm netting cages and provided with 6% glucose solution *ad libitum* at 25-28°C and a relative humidity from 68-75%. Saturated cotton towels (50x25cm) were folded and placed over the cages to avoid mosquito desiccation. Mosquitoes were starved from sugar for 7 h and allowed to feed on a human arm for 15 minutes at 19.00h on the same day the same procedure was repeated 24 hours later. After the first blood meal unfed mosquitoes were removed from the cages. Fed mosquitoes were kept together with males for two more days after the second blood meal before they were utilized in an experiment (i.e. females aged 4–5 days after first blood meal). Host-seeking mosquitoes were prepared by selecting 300 two to three days old females on the day of experiment. Mosquitoes were starved for 6 h before the experiment commenced at 18.00h.

### Experimental design

All experiments were implemented in a single semi-field system. Two hundred gravid female mosquitoes were selected from the holding cages based on their abdominal stages (whitish in colour and oval in shape) and were released into the semi-field system between 17.30h and 18.00h. Experiments were terminated at 08.00h the following morning. Experiments with more than one treatment followed a randomized complete block design. Treatments were assigned randomly (using a random number generator) to the corners of the semi-field system and rotated randomly across corners until all treatments were run once in each of the corners included in the respective experiment. This block of experiments was then repeated. Experiments were carried out for 8 or 12 nights.

### Experiments

#### Evaluation of two spark box settings to optimize mosquito collections with e-nets

While e-nets hold promise for studying mosquito behaviour there are a few potential problems that needed to be investigated. High spark energy is used for collecting large insects like tsetse flies [27-29], however, such high energy makes the net spark, creating a crackling sound, a burst of light and a burning smell, that may affect mosquito movement or it may destroy them by burning. Therefore, a modified transformer was used which allowed the moderation of the voltage to eliminate the sparking. Nevertheless, reduced sparking might also allow mosquitoes to escape. Accordingly, an experiment was designed to compare the catches of e-nets powered by a low-power or standard transformer.

This experiment was done using unfed *An. gambiae* s.s. females since previous research using e-nets used mosquitoes of this physiological stage and therefore a reliable response towards the target was expected [27,28]. All consequent experiments were done with gravid females. Two e-nets were positioned in opposite corners of a semi-field system. E-nets were mounted over water-filled trays (45×85×6cm) that served to collect stunned mosquitoes that fell to the ground [27,28]. An odour source of carbon dioxide and a cotton sock worn for 8 hours was used as an attractant [43-46] and positioned on the opposite side of the e-net, 70cm from the e-nets and corner walls of the semi-field system. Two power settings on the spark boxes were compared: 100% spark energy which produced sparks and 50% spark energy which was the highest energy setting that did not produce sparks. The experiment was carried out for 8 nights.

### Assessment of sticky boards as collection device under e-nets

A second problem associated with e-nets is how to collect the stunned mosquitoes. Insects killed or stunned after colliding with the e-net fall to the ground. For ease of collection and to prevent them from being eaten by ants and other predators a catching device on the ground was needed. Water-filled trays under the e-nets worked well in experiments with host-seeking mosquitoes [27,28], however when studying the behaviour of gravid mosquitoes, water-filled trays cannot be used since they might attract gravid mosquitoes in search of an oviposition site and divert them from the intended target. A series of experiments with e-nets positioned over sticky boards were carried out to find the most suitable material for collecting mosquitoes when stunned by the electric net.

### Evaluation of cardboard mounted with transparent sticky films

One e-net was set up in a corner of a semi-field system (Sites 1–4) and a round pond placed 70 cm from the e-net, between the net and the corner to attract gravid females. Transparent sticky film was mounted on two 50x80cm cardboard rectangles. A grid of two rows, each row with 4 cells (20x25cm), was marked on the boards. One board was placed on each side of the e-net (Figure 3A). The e-net was charged using 50% spark energy and the experiment carried out for 8 nights. The number of mosquitoes that got stuck on the film was counted separately for each cell and direction towards the e-net.

### Evaluation of potential attraction of gravid *An. gambiae* s.s. towards transparent sticky films

A collection device under an e-net should not attract gravid mosquitoes otherwise the number of mosquitoes approaching a target will be overestimated. Shiny sticky surfaces may, to a gravid mosquito, look like a water body. Accordingly, studies were undertaken to assess whether gravid mosquitoes landed on the shiny surfaces of the transparent sticky films. Four boards (50×80cm) were prepared with transparent sticky film. Two of the boards were placed on the ground in one corner and the other pair of boards in the opposite corner of the semi-field system. In order to test if landing on the sticky boards is associated with a resting behaviour close to a water source just prior or after egg-laying or if it is an actual attraction towards the surface an artificial pond was added to one of the two treatments. A round artificial pond was dug into the sand 20cm behind one of the pairs of the sticky boards. The experiment was carried out for 8 nights. The number of mosquitoes that landed on the boards was recorded.

### Comparison of yellow, black and transparent film sticky boards for the collection of gravid *An. gambiae* s.s

**Figure 4:**
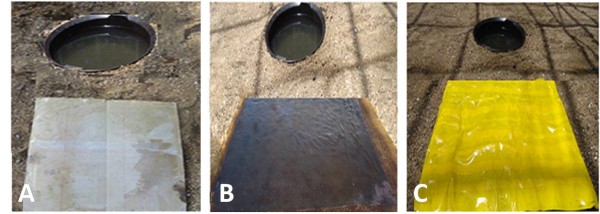
**Three sticky boards evaluated in comparison to assess the attraction of gravid mosquitoes towards their surfaces: (A) transparent sticky film, (B) sticky black fly-screen, (C) yellow sticky film**.

To find a non-attractive device for the collection of electrocuted mosquitoes, three sticky surfaces different in texture and colour were compared. Three cardboard squares of 50x50cm were covered with one of the following treatments (Figure 4): (1) transparent sticky film; (2) black netting painted with 100 g insect glue dissolved in 25 ml hexane; and (3) yellow sticky film. Boards were positioned in three different corners of the semi-field system. One corner remained empty but was included in the random allocation of treatment location. Round artificial ponds were dug into the sand at a distance of 20cm behind each of the boards (Figure 4).The experiment was carried out for 12 nights. The number of mosquitoes that landed on the boards and the number of eggs laid in their respective ponds were recorded.

### Collection efficacy of a square of e-nets surrounding an artificial oviposition site

**Figure 5:**
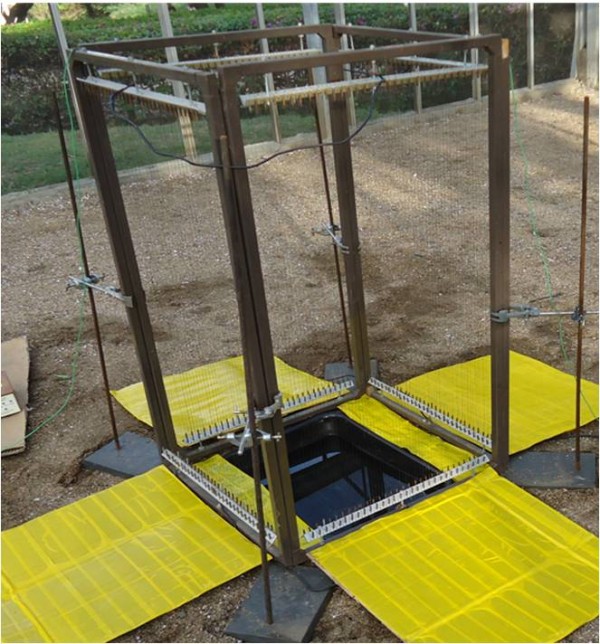
**A complete square of four electrocuting nets surrounding an artificial aquatic habitat.** Yellow sticky boards serve as collection device for stunned mosquitoes.

A complete square of four e-nets was mounted around a rectangular pond set up in the centre of the semi-field system in order to estimate the number of gravid females approaching water. Adjacent e-nets were held together by clamps on stands and two of them shared one battery and a spark box (Figure 5). E-nets were charged with 50% spark energy. Four yellow sticky boards of 50×50cm were placed in front of each of the e-nets. Any open space inside the square of e-nets was also covered with yellow sticky board (Figure 5). Boards were divided into two horizontal rows (25×50cm) for further evaluation of the efficacy of the net and of the board as a collection device. The number of mosquitoes collected in the two rows of the board and inside the square of e-nets was recorded separately. Any eggs in the ponds were counted.

### The use of sticky materials and detergents to assess if and where *An. gambiae* s.s. land on aquatic habitats when laying eggs

A prerequisite for the development of new monitoring control tools targeting oviposition site seeking mosquitoes e.g. with gravid traps [18-20,38,47-53] is to know if and where gravid females land during oviposition. Very few studies have assessed this particular behaviour and a variety of different modes of oviposition have been described. Here the use of different sticky materials and a detergent are evaluated to analyse potential landing of gravid females on the water surface or habitat edge for laying eggs.

### Assessment of landing on the habitat edge

The edges of three round artificial ponds were made sticky to trap any landing mosquito by applying one of the three treatments: (A) yellow sticky film, (B) spray glue or (C) transparent double-sided sticky film to their inner walls. The sticky edge was 7cm wide and bordered the water surface. The ponds were set up in three corners of a semi-field system. The empty corner was included in the randomization of the treatments. The experiment was run for 12 nights. The number of mosquitoes stuck to the sticky edges and the number of eggs laid in the ponds were recorded.

### Assessment of landing on the water surface

**Figure 6:**
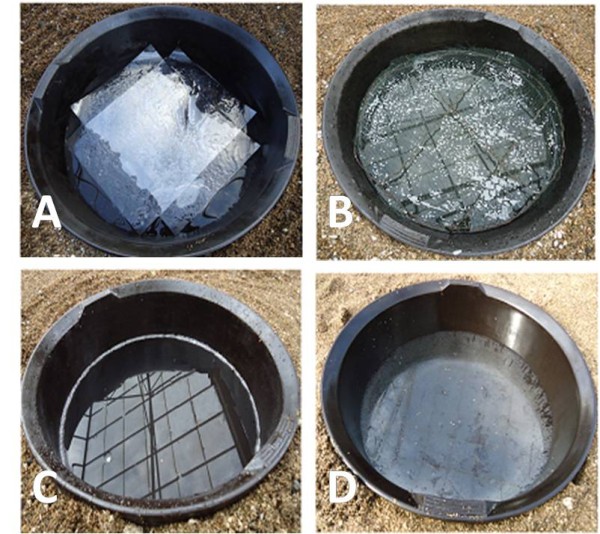
Surface treated artificial ponds with (A) sticky transparency, (B) sticky wire screen (C) detergent, (D) spray glue.

Four round artificial ponds were prepared. One of the following four treatments were applied on the water surfaces: (1) two A4 overhead projection transparencies were overlain on each other with colourless adhesive tape to form a cross-shaped surface; the transparencies were coated on one side with 100 g insect glue dissolved in 30 ml hexane and placed on the water surface leaving 8areas of approximately 105cm^2^ free water access at the edges (Figure 6A); (2) a circle of dark-green wire screen of the same area as the pond was prepared and coated with 100g insect glue dissolved in 30ml hexane; the wire screen was mounted on a square of wire and placed horizontally inside the pond 5cm below the edge of the pond and 2cm above the water surface (Figure 6B); (3) 225ml (2.5%) detergent was added to the water (Figure 6C); (4) insect spray glue was uniformly sprayed on the water surface (Figure 6D). Ponds were set up in the corners of the semi-field system and the experiment carried out on 12 nights. The number of mosquitoes caught and the number of eggs laid in each pond were recorded.

### Evaluation of the landing behaviour using a combination of detergent in the water and spray glue on the edge of the pond

Finally the best catching tools from the previous two experiments were combined, to assess whether there is a sequence in the landing behaviour (e.g. landing on surface for egg-laying and then resting on the edge of the pond). One round artificial pond was prepared. On the edge spay glue was applied and 225ml detergent added to the water. The artificial pond was set up in the centre of a semi-field system. The experiment was carried out for 8 nights. The number of mosquitoes caught and the number of eggs in the pond were recorded.

### Data analysis

The data generated in this study were count data, i.e. either the number of gravid mosquitoes recollected or the number of eggs laid in artificial ponds, and were not normally distributed. Therefore, multivariable analyses were done with untransformed data using generalized linear models [54,55]. Data analyses were done with R statistical software version 2.13.1, including the contributing packages MASS, effects, epicalc, multcomp, lme4, gee, geepack, aod [56]. Experiments with one treatment tested in the semi-field system each day were analysed using generalized linear models with negative binomial data distribution using the glm.nb function and a log link function. Data collected for two or more treatments in the same semi-field system on the same day were not independent and were therefore analysed with generalized estimating equations using the gee or geepack function. In this case the repeated measure was the day of experiment. Here, a Poisson distribution of the data was used in the model and an exchangeable working correlation matrix. The fixed factor variables included in this model were the treatments of interest and the corner of the semi-field system (site) in which a treatment was placed. It was thought possible that the probability of catching mosquitoes might differ between the four corners (sites 1–4) of the greenhouse, independently from the test treatment, due to slightly different environmental factors such as light intensity, wind direction and microclimate. If the effect of site was insignificant this variable was removed from the final model. The output presented in the tables includes only significant factors from the final model.

The parameter estimates of the models were used to predicted the mean counts per treatment and their 95% confidence intervals (CIs) by removing the intercept from the models [54]. Similarly, multiple comparisons of treatments were calculated based on the model parameter estimates.

## Results

### Evaluation of two spark box settings to optimize mosquito collections with e-nets

With the low energy setting, twice as many *An. gambiae s.s*. mosquitoes were collected than with the high energy setting (Table 2). Thus, the low energy setting was chosen for all subsequent experiments with gravid females.

**Table 2:** **The development of electrocuting nets as a tool to study the orientation behaviour of oviposition site seeking *An. gambiae *s.s**

**Treatment**	**Mean no. of mosquitoes/eggs (95% CI)**	**OR (95% CI)**	**p**
**Experiment: Evaluation of high and low energy settings for electrocuting nets**			
100% spark energy	9.0 (5.9 – 13.7)	1	
50% spark energy	19.9 (13.2 – 30.0)	2.2 (1.8 – 2.8)	<0.001
**Experiment: Comparison of average mosquitocollections on transparent sticky film boards close and away from one e-net**			
row 2 (>25 cm)	26.5 (16.15 – 43.49)	1	
row 1 (<25 cm)	77.63 (62.36 – 96.63)	2.93 (2.08 – 4.13)	< 0.001
**Experiment: Evaluation of attraction of gravid *An. gambiae *s.s. to transparent sticky films**			
without pond	29.4 (21.5 – 40.4)	1	
with pond	47.1 (37.4 – 59.3)	1.6 (1.1 – 2.3)	0.012
site 4	15.3 (11.2 – 20.7)	1	
site 2	38.4 (30.0 – 49.1)	2.5 (1.6 – 4.1)	<0.001
**Experiment: Comparison of yellow, black and transparent sticky boards for the collection of gravid *An. gambiae *s.s.**			
Mosquitoes*			
Transparent sticky film	24.6 (18.4 –32.9)	1^a^	
Sticky black fly-screen	17.3 (12.0 –25.1)	0.71 (0.39 – 1.26) ^a^	0.240
Yellow sticky film	0.58 (0.32 –1.09)	0.02 (0.01 – 0.05) ^b^	<0.001
Eggs*			
Transparent sticky film	478 (356 – 643)	1^a^	
Sticky black fly-screen	469 (326 – 469)	0.98 (0.80 – 1.20) ^a^	0.841
Yellow sticky film	712 (525 – 712)	1.50 (1.18 – 1.92) ^b^	0.001
**Experiment: Comparison of average mosquito collections on yellow sticky film boards mounted under a square of e-nets**			
row 2 (>25 cm)	5.1 (3.9 – 6.8)	1	
row 1 (<25 cm)	48.1 (40.7 – 56.9)	9.4 (7.7 –11.4)	<0.001
inside the square	12 (9.6 – 15.0)	2.3 (1.8 – 3.0)	<0.001

Results from generalized linear models for individual experiments.

CI=confidence interval.

OR=odds ratio.

*Multiple comparisons of treatments: treatments denoted with same letter are not significantly different.

### Evaluation of cardboards mounted with transparent sticky films

**Figure 7:**
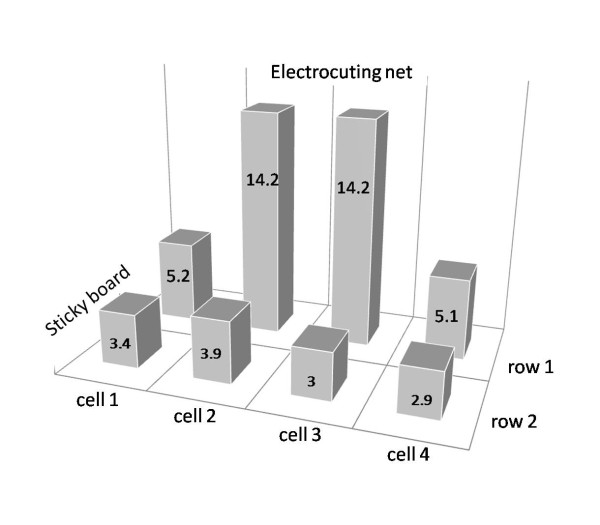
**Distribution of electrocuted mosquitoes on the transparent sticky film collection board.** The height of the columns show the average number of mosquitoes collected per cell of the grid drawn on the board.

In the first e-net experiment with gravid females, an average of 104.1 females (95% CI 78.0-138.9) were collected per night on the transparent film of the collection boards, representing around 50% of females released. Similar numbers were caught on both sides of the e-net, with greatest numbers close to the net in the centre (Table 2, Figure 7). This distribution indicated that most mosquitoes were electrocuted by the net but many females on the row furthest from the e-net appeared to ‘sit’ on the board rather than lay on the side as was the case when stunned, some were even still alive in the morning. This suggested that some females were not stunned by the net but had been attracted by the shiny film and landed on it. If this was true the number of mosquitoes on the collection board overestimated the number attracted by the water and stunned by the e-net. It was, therefore, necessary to evaluate the potential attractiveness of the collection device in the next experiments.

### Evaluation of attraction of gravid *An. gambiae s.s*. to transparent sticky films

In this experiment mosquito collections were significantly affected by the corner in which the treatments were presented in the semi-field system. If any of the two treatments was set up in site 2 it was 2.5 times more likely to catch a mosquito than if it was set up in site 4 (Table 2). Adjusting for corner, the analyses showed that the sticky board alone caught approximately 15% of the released mosquitoes, while 24% were collected when the sticky board was placed next to water. These results suggest that the sticky board alone was attractive to gravid females and their landing on it was not associated with resting around a potential habitat otherwise females should not have been trapped by the boards without pond. This experiment also confirms that water vapour is a strong attractant for oviposition site seeking mosquitoes.

### Comparison of yellow, black and transparent sticky boards for the collection of gravid *An. gambiae* s.s

**Figure 8:**
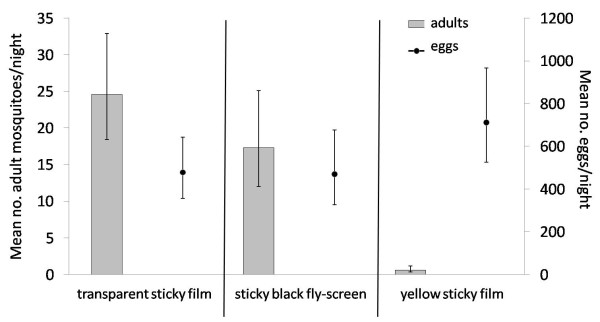
**Mean number (error bars: 95% confidence intervals) of gravid females collected on three types of sticky boards and the mean number of eggs laid in the ponds associated with the boards**.

Gravid females were equally attracted by the transparent sticky film and the sticky black fly-screen, yet few were collected on the yellow sticky film. Furthermore, a significantly higher number of eggs were laid in the pond behind the yellow boards than in the ponds behind the other sticky materials (Table 2, Figure 8). Yellow sticky boards did not interfere with the approach of the gravid female towards a pond and consequent egg-laying and were therefore chosen as routine collection device under e-nets.

### Collection efficacy of a square of e-nets around an artificial pond

In order to estimate the number of gravid females approaching an aquatic habitat a complete square of e-nets was used that surrounded an artificial pond (Figure 5). On average one third (65.3 (95% CI 55.9 – 76.10)) of the 200 released mosquitoes were collected. Over 81% of these were found on the outside of the ring indicating that only few gravid females might have approached the oviposition site from a height above 1m from the ground or passed through the 8 mm gaps between the vertical aluminium frames and the wires. Low numbers of eggs were found on 6 out of 8 days. The average number of eggs was 80.3 (95% CI 43.6 – 147.8). On average over nine times as many mosquitoes were found on the sticky board close to the e-nets than further away suggesting that they were stunned by the electric nets and hence fell close to the base of the net (Table 2).

### The use of sticky materials and detergents to assess if and where *An. gambiae* s.s. land on aquatic habitats when laying eggs

On average the number of females trapped on the water surfaces was over four times higher than on the edges (103.3, 95% CI 93.0-115 and 23.7, 95% CI 20–28.2, respectively) irrespective of collection device (Tables 3 and Figure 8). The detergent and the spray glue caught about three times more mosquitoes than the sticky wire screen or transparencies (Table 3). The detergent lowered the water surface tension to such an extent that mosquitoes that landed on the water surface sunk, presenting little opportunity to lay eggs. On the other hand, a large proportion of the mosquitoes stuck on the surface with spray glue laid eggs, leading to more than 11 times higher mean egg numbers than other treatments (Table 3).

**Table 3:** **The use of sticky materials and detergents to assess if and where *An. gambiae *s.s. land on aquatic habitats when laying eggs**

**Treatment**	**Mean no. of mosquitoes/eggs (95% CI)**	**OR (95% CI)**	**p**
**Experiment: Assessment of landing and egg-laying on the habitat surface**			
**Mosquitoes**			
wire screen	11.9 (8.1 –17.6)	1^a^	
spray glue	35.2 (27.4 – 45.1)	3.0 (2.1 – 4.1) ^b^	<0.001
detergent	41.7 (32.6 – 53.2)	3.5 (2.0 – 6.1) ^b^	<0.001
transparency	14.6 (10.8 – 19.7)	1.2 (0.7 – 2.0) ^a^	0.460
site 1	20.4 (14.3 – 29.2)	1^a^	
site 2	16.4 (12.5 – 21.6)	0.8 (0.6 – 1.0) ^a^	0.070
site 3	37.5 (26.1 – 54.0)	1.8 (1.3 – 2.5)^b^	<0.001
site 4	29.0 (21.0 – 40.0)	1.4 (1.2 – 1.7) ^b^	<0.001
**Eggs**			
wire screen	39 (23–65)	1^a^	
spray glue	464 (344 – 628)	11.9 (6.8 – 20.9) ^b^	<0.001
detergent	12 (4 – 34)	0.3 (0.1 – 0.8) ^c^	0.018
transparency	23 (10 – 52)	0.6 (0.3 – 1.1) ^ac^	0.109
site 1	105 (42 – 259)	1^a^	
site 2	79 (29 – 219)	0.8 (0.4 – 1.6) ^a^	0.546
site 3	173 (68 – 439)	1.8 (1.1 – 3.1) ^b^	0.026
site 4	181 (79 – 419)	1.8 (1.3 – 2.5) ^b^	0.001
**Experiment: Assessment of landing on the habitat edge for egg-laying**			
**Mosquitoes**			
spray glue	13 (9.4 – 18.0)	1^a^	
yellow sticky film	5.4 (3.0 – 10.0)	0.4 (0.2 – 0.8) ^b^	0.012
transparent double-sided sticky film	5.3 (4.0 – 7.3)	0.4 (0.2 – 0.6) ^b^	<0.001
site 1	4.9 (3.1 – 7.7)	1^a^	
site 2	10.8 (6.7 – 17.5)	2.5 (1.5 – 4.1) ^b^	<0.001
site 3	9.0 (6.0 – 13.6)	1.9 (1.0 – 3.5)^b^	0.036
site 4	7.1 (4.1 – 12.2)	1.7 (0.8 – 2.9) ^a^	0.062
**Eggs**			
spray glue	358 (232 – 552)	1^a^	
yellow sticky film	363 (240 – 549)	1.0 (0.6 – 1.7) ^a^	0.930
transparent double-sided sticky film	297 (178 – 497)	0.8 (0.5 – 1.4) ^a^	0.420
**Experiment: Evaluation of sequence of landing on habitat during oviposition**			
surface catch (detergent)	42.5 (37.7 – 47.9)	1	
edge catch (spray glue)	7.4 (5.1 – 10.8)	0.17 (0.12 – 0.25)	<0.001

Results from generalized linear models for individual experiments.

CI=confidence interval.

OR=odds ratio.

Multiple comparison of treatments: treatments denoted with same letter are not significantly different.

From those treatments applied to the edge of the pond, the yellow and transparent films trapped similar numbers of mosquitoes but less than half of the spray glue (Table 3). Similar egg numbers in all the treatments indicate that a similar number of gravid females approached these ponds and laid eggs. It is unlikely that all these eggs were laid by the few mosquitoes trapped on the edge. The mean number of eggs in these ponds is comparable with the mean number laid by mosquitoes stuck on the spray glue on the water surface (Table 3).

Finally, when detergent in the water was combined with spray glue on the edge of a pond, most mosquitoes were drowned in the water with only 15% stuck on the pond edge (Table 3). Notably, approximately a quarter of the released mosquitoes were collected with this method. This is only slightly less than the figures obtained from the square of e-nets where approximately one third of all released mosquitoes were collected. Eggs were not found in the pond throughout the test nights suggesting that oviposition did not take place in flight.

## Discussion

Electric nets have been used successfully for the development of control tools for tsetse flies for nearly 40 years [23,29,57], yet have been used little for mosquito research [27,28]. Results presented here show that e-nets can be used to study the oviposition behaviour of malaria vectors. Importantly, it was found that reducing the voltage to prevent sparking doubled the catch, which confirms earlier findings by Torr and colleagues [28]. It is uncertain whether it is the visual, acoustic or chemical cues associated with the sparking that reduces the catch. When a single e-net was used next to an artificial pond, similar numbers of mosquitoes were collected on both sides of the net indicating that the mosquitoes approached the target from both directions. In order to quantify the total number approaching an attractive source, such as a water body, a complete square of e-nets surrounding the water was found useful. Field tests need to evaluate the performance of the e-nets for studying gravid mosquitoes under open field conditions, especially during rainy seasons the normal periods of maximum malaria transmission. Previous work on host-seeking *An. arabiensis* has shown that e-nets covered with a small roof work well even when it rains [28].

Sticky boards proved to be a simple method for collecting mosquitoes that were stunned after colliding with the net and fell to the ground since they effectively retained specimens and protected them from predation by ants. However, it was found that a transparent film was also attractive to gravid mosquitoes, even when used as sole collection device without any e-nets and without a water source nearby. Adding an artificial pond behind the transparent film sticky board increased the number of females trapped on the board confirming that water vapour is a strong attractant for oviposition site seeking mosquitoes [40,58,59].

In search of an alternative collection material under e-nets, the black fibreglass gauze coated with insect glue proved as attractive to gravid mosquitoes as transparent film. Both surfaces were conspicuously shiny for the human eye compared to the yellow film that appeared matt and might act as a visual cue for gravid females. Previously, black flies of all physiological stages have been successfully trapped with glue coated aluminium plates [60-62] and in a recent study, Harris and colleagues [63] utilized this principle to collect gravid mosquitoes from water surfaces using glue-coated transparencies. Many insects, including mosquitoes, respond to reflectance of water surfaces to locate water bodies to lay their eggs, often using horizontally polarized light reflected from the water surface as orientation cues [64-69]. Surfaces with high polarized light reflectance might be promising as trapping devices alone or in combination with a gravid trap for monitoring African malaria vectors. Nevertheless, it can not be excluded that a chemical cue associated with the insect glue attracted the gravid females and there is need to further investigate the properties of the glue-coated surfaces used in this study.

The low number of mosquitoes on the yellow sticky film and the high number of eggs laid in the adjacent pond suggest that this material does not have the same visual properties for a mosquito as the transparent film and black glue boards and does not attract mosquitoes. Oviposition site-seeking females fly straight to the pond to lay their eggs, and then fly off again, without landing close to the aquatic habitat before or after egg-laying. This might be due to the light colour [70] and the lack of reflectance. It is unlikely that it has to do with the actual colour of the board since mosquitoes have dichromatic vision, which results in good contrast sensitivity but poor colour resolution [71]. It is known that mosquitoes respond to contrasts [64,70] and gravid females are attracted by dark surfaces rather than light coloured ones [64].

The number of mosquitoes collected with transparent sticky boards was approximately twice the number collected with yellow sticky boards. It is likely that transparent films overestimated the number of mosquitoes that approached the pond when they were used in combination with e-nets but sticky boards made of the yellow film can serve as effective collection device. On the other hand, the attractiveness of the boards mounted with transparent sticky film might be exploited further in future for the development of new trapping devices for gravid malaria vectors.

For the development of new interventions (e.g. auto-dissemination of larvicides [72-74]) and monitoring tools (e.g. ovi-traps and gravid traps [18-20,38,47-53]) targeting gravid malaria vectors it is important to know if and where gravid females land during oviposition. Notably, very few studies have investigated this and all these studies used relatively small cages (less than 1m^3^) except one which was implemented under field conditions [63]. Gravid females were most commonly observed laying their eggs directly, either laying eggs when on the water surface or on the lip of the oviposition cup [59,64,75]. Occasionally, oviposition from flight has been described when the oviposition cup was placed over a black surface [64]. Here, for the first time, experiments in large semi-field systems are described that investigate if and where *An. gambiae* s.s. lands to lay her eggs. The results indicate that gravid females primarily land directly on the water surface to lay eggs. Since no eggs were found in ponds with both detergent and sticky sides, which prevents directly egg-laying on the water surface, there is no evidence for eggs being dropped in flight onto the water from these experiments. The relatively large number of eggs found associated with females caught on the spray glue applied on the water surface was probably due to stress induced oviposition on the surface [63].

Similar numbers of eggs were laid in ponds treated with different sticky materials at their edges, though the number of adults caught on the edges differed, the number of adults caught there was small. This suggests that even mosquitoes caught at the edge might have landed there to rest before or after laying eggs, rather than to lay whilst seated on the edge of the pond. In the case of the pond with spray glue at the edge attraction of female mosquitoes cannot be excluded since the numbers were significantly higher than for the other two treatments and the glue made the pond edge appear very shiny.

Some caution must be exercised when interpreting the data since the artificial ponds used in this study had a sharp vertical edge which was not utilized by gravid females to sit on and lay eggs. This might have been different if ponds with a slope would have been used. Previous cage experiments have shown that *An. gambiae* s.s. and *Anopheles arabiensis* laid a large proportion of eggs on water saturated slopes rather than the free-standing water when given a choice [58,75,76]. Nevertheless, even then it was observed that these eggs were laid whilst on the water surface rather than during flight [75].

The finding of this study that *An. gambiae* s.s. lays its eggs directly on the water surface supports the observations made on *An. arabiensis* by Harris and colleagues [63] in the field using transparencies floating on the water on the edge of natural habitats. The finding that gravid *An. gambiae s.s.* lay their eggs directly on the water surface is encouraging for two reasons. Firstly, it lends support to the principle that gravid females could be used to transfer larvicides from a resting site to a breeding site [72-74,77]. Secondly, it may lead to the development of a gravid trap where mosquitoes are attracted to a water source and trapped there [20].

Sticky materials and the detergent used in this study were shown to be useful methods for collecting mosquitoes when landing to lay eggs. Of all the tools tested the detergent and the spray glue directly applied to the water surface was most effective at collecting gravid females under semi-field conditions. Transparencies and sticky screens did not work as well which might be due to obstruction of water vapour coming off the pond by the transparency or due to visual obstruction of the water surface area. The latter two might have been useful tools for testing the attraction of female vectors towards a water source that was treated with putative oviposition semiochemicals or natural infusions [40] but due to their reduced trapping efficiency e-nets might be the best alternative for analysing such odour-oriented behaviour. Detergents and spray glue, though powerful in arresting approaching females, might interfere with the presented chemical or infusion. Therefore, further research would be required to present these in combination for attracting and trapping gravid female mosquitoes. The use of these tools under natural conditions also needs to be further evaluated.

## Conclusion

This study demonstrated that electric grids are suitable devices for studying the egg-laying behaviour of *An. gambiae s.s.* when used in combination with yellow sticky boards for collecting stunned mosquitoes. Shiny sticky surfaces attract gravid females possibly because they are visually mistaken as breeding sites. These materials might be developed further as gravid traps. *Anopheles gambiae* s.s. primarily land on the water surface for oviposition. This behaviour can be exploited for the development of new trapping and control strategies.

## Competing interests

The authors declare that they have no competing interests.

## Authors’ contributions

UF, JL and ST conceived the idea for this research. UF, JL, SD and ST developed the experimental design and SD developed all protocols and implemented the experiments. SD and UF analysed the data and drafted the manuscript. SWL and FO contributed to the development of the protocols. All authors contributed to the final draft, read and approved the manuscript.
